# Red cell distribution width is a potent prognostic parameter for in-hospital and post-discharge mortality in hospitalized coronavirus disease 2019 patients: a registry-based cohort study on 3941 patients

**DOI:** 10.3325/cmj.2022.63.44

**Published:** 2022-02

**Authors:** Marko Lucijanić, Ana Jordan, Ivana Jurin, Nevenka Piskač Živković, Ena Sorić, Irzal Hadžibegović, Armin Atić, Josip Stojić, Diana Rudan, Ozren Jakšić, Nikolina Bušić, Lovorka Đerek, Ivica Lukšić, Bruno Baršić

**Affiliations:** 1 Hematology Department, University Hospital Dubrava, Zagreb, Croatia; 2 Primary Respiratory and Intensive Care Center, University Hospital Dubrava, Zagreb, Croatia; 3 University of Zagreb, School of Medicine, Zagreb, Croatia; 4 Cardiology Department, University Hospital Dubrava, Zagreb, Croatia; 5 Pulmonology Department, University Hospital Dubrava, Zagreb, Croatia; 6 Faculty of Dental Medicine and Healthcare, Josip Juraj Strossmayer University, Osijek, Croatia; 7 Department of Emergency Medicine, University Hospital Dubrava, Zagreb, Croatia; 8 University North, University Center Varaždin, Varaždin, Croatia; 9 Catholic University of Croatia, Zagreb, Croatia; 10 Clinical Department for Laboratory Diagnostics, University Hospital Dubrava, Zagreb, Croatia; 11 Department of Maxillofacial Surgery, University Hospital Dubrava, Zagreb, Croatia

## Abstract

**Aim:**

To investigate clinical and prognostic associations of red cell distribution width (RDW) in hospitalized coronavirus disease 2019 (COVID-19) patients.

**Methods:**

We retrospectively analyzed the records of 3941 consecutive COVID-19 patients admitted to a tertiary-level institution from March 2020 to March 2021 who had available RDW on admission.

**Results:**

The median age was 74 years. The median Charlson comorbidity index (CCI) was 4. The majority of patients (84.1%) on admission presented with severe or critical COVID-19. Patients with higher RDW were significantly more likely to be older and female, to present earlier during infection, and to have higher comorbidity burden, worse functional status, and critical presentation of COVID-19 on admission. RDW was not significantly associated with C-reactive protein, occurrence of pneumonia, or need for oxygen supplementation on admission. During hospital stay, patients with higher RDW were significantly more likely to require high-flow oxygen therapy, mechanical ventilation, intensive care unit, and to experience prolonged immobilization, venous thromboembolism, bleeding, and bacterial sepsis. Thirty-day and post-hospital discharge mortality gradually increased with each rising RDW percent-point. In a series of multivariate Cox-regression models, RDW demonstrated robust prognostic properties at >14% cut-off level. This cut-off was associated with inferior 30-day and post-discharge survival independently of COVID-19 severity, age, and CCI; and with 30-day survival independently of COVID severity and established prognostic scores (CURB-65, 4C-mortality, COVID-gram and VACO-index).

**Conclusion:**

RDW has a complex relationship with COVID-19-associated inflammatory state and is affected by prior comorbidities. RDW can improve the prognostication in hospitalized COVID-19 patients.

Coronavirus disease 2019 (COVID-19), caused by severe acute respiratory syndrome coronavirus-2 (SARS-CoV-2), is a systemic infectious disease usually presenting with fever and respiratory symptoms (1). Although the most frequent serious manifestation of COVID-19 is pneumonia, the disease has been associated with cardiovascular, neurological, and gastrointestinal symptoms (2). Systemic inflammatory response mediated by high interleukin-6 concentrations induced by SARS-CoV-2 infection is associated with more severe clinical presentation, respiratory deterioration, and death (3,4). The presence of prior chronic comorbidities substantially affects the survival of COVID-19 patients (1).

Anisocytosis, ie, unequal red blood cells (RBC) size, is a sensitive marker of distress in erythropoiesis or RBC destruction. It can be induced by various metabolic and inflammatory stimuli, nutrient deficiencies, infections, spleen disorders, and specific drugs interfering with RBC production (5). Anisocytosis can be quantified as a coefficient of variation of mean cell volume termed red blood cell distribution width (RDW), which is obtained by automatic cell counters. Higher RDW levels have recently gained attention as they are uniformly associated with unfavorable presentation and inferior outcomes in many chronic metabolic and malignant diseases (6-12). More severe clinical presentation and higher mortality rates were also found in COVID-19 patients with higher RDW levels (13-16). However, an association of RDW with other clinical outcomes in hospitalized COVID-19 patients, as well as the relationship with increased mortality in the context of other established prognostic scores, are not well defined. Thus, we aimed to investigate clinical and prognostic significance of RDW in a large cohort of hospitalized COVID-19 patients from our institution. We hypothesized that RDW was associated with more severe COVID-19 on admission and higher death rate.

## Patients and methods

We retrospectively analyzed the records of 3941 patients admitted to University Hospital Dubrava because of acute COVID-19 infection from March 2020 to March 2021. We included only patients who had available data on RDW on admission. During the pandemic, our institution was repurposed into a tertiary COVID-19 regional center treating patients with the most serious COVID-19 clinical presentation and COVID-19 patients with comorbidities who required immediate hospital level of care. All patients had a positive polymerase chain reaction or rapid antigen COVID-19 test before hospital admission. Patients were treated according to the contemporary guidelines. Only index hospital admissions for acute COVID-19 were investigated. Data were obtained from the hospital registry thorough an analysis of electronic and written medical records of 4014 COVID-19 patients. Seventy-three patients were excluded due to lack of available RDW data on admission. The study was approved by the University Hospital Dubrava Review Board (2021/2503-04).

RDW on admission was expressed as a coefficient of variation (%) of mean corpuscular volume (MCV) as reported by Advia 2120i automated cell counter (Siemens Medical Solutions Diagnostics Pte Ltd, Swords, Ireland). COVID-19 severity at admission was graded according the World Health Organization (WHO) recommendations and national guidelines (17,18) as mild, moderate, severe, and critical. Comorbidities, assessed as individual entities, were summarized by using the Charlson comorbidity index. Mortality and other clinical outcomes were assessed from the start of hospital stay.

Modified early warning score (MEWS) was used to quantify COVID-19 symptoms. Eastern Cooperative Oncology Group (ECOG) classification was used to estimate functional status on admission. Confusion, urea, respiratory rate, blood pressure, and 65 years of age (CURB-65); International Severe Acute Respiratory and emerging Infections Consortium 4C mortality score; COVID-gram; and Veterans Health Administration COVID-19 (VACO) index were used as prognostic risk scores. Chronic Kidney Disease Epidemiology Collaboration (CKD-EPI) formula was used for the calculation of estimated glomerular filtration rate (eGFR) .

The primary objective was to assess the clinical associations of RDW in the context of acute COVID-19 and its prognostic properties for investigated clinical outcomes. The secondary objectives were to assess the relationship with baseline clinical characteristics (both demographic and COVID-19-related), laboratory and radiologic features, and to establish appropriate optimized RDW cut-off levels to discriminate patient groups according to the risk of unwanted outcomes (30-day mortality, need for high flow oxygen therapy, need for mechanical ventilation, need for intensive care unit, arterial thromboses, venous thromboses, bleeding complications, bacterial sepsis, six-month post hospital discharge mortality, hospital-readmission).

### Statistical methods

The normality of distribution of numerical variables was assessed with the Shapiro-Wilk test. The results are presented as median and interquartile range (IQR), and the groups were compared by using the Mann-Whitney U test or Kruskal-Wallis one-way analysis of variance (ANOVA) where appropriate. Jonckheere-Terpstra test for trend was used to assess the trend of increase in interleukin-6 concentrations across RDW categories. The receiver operating characteristic (ROC) curve analysis was used to define optimal cut-off values of RDW for clinical outcomes. Categorical variables are presented as frequencies and percentages, and were compared between the groups by using the Χ^2^ test or the Fisher test where appropriate. Survival analyses were based on the Kaplan-Meier method. For univariate survival analyses, the Cox-Mantel version of the log-rank test and the Cox regression analysis were used. For multivariate analyses, the Cox regression analysis was used while simultaneously controlling for all included variables. *P* values <0.05 were considered statistically significant. All analyses were performed with MedCalc statistical software, version 20.008 (MedCalc Software Ltd, Ostend, Belgium).

## Results

### The association of patients' characteristics and RDW

The study enrolled 3941 hospitalized COVID-19 patients with available RDW values on admission (2215 or 56.2% men). The median age was 74 years; the median Charlson comorbidity index was 4 (IQR 3-6). The majority of patients (3316 or 84.1%) presented with severe or critical COVID-19 symptoms. During hospital stay, 901 (22.9%) patients required intensive care unit treatment, 768 (19.5%) required high-flow oxygen therapy, and 669 (17%) required mechanical ventilation. Thirty-day survival was 65.6%.

The median RDW was 14.1% (IQR 13.4-15.2). The optimal cut-off level for in-hospital and post-discharge survival discrimination defined by the ROC curve analysis was >14%. Patients’ characteristics stratified at this level are presented in Table 1. Patients with higher RDW were significantly more likely to be older, to be female, to present earlier in the disease course, and to have a worse functional status on admission. Patients with higher RDW were more likely to be admitted to hospital due to an acute medical or surgical condition as a leading reason and were less likely to be admitted due to pneumonia itself. Although patients with higher and lower RDW had a similar frequency of radiological pneumonia and need for oxygen supplementation, patients with higher RDW were more likely to have critical severity of symptoms and to have other infection on admission (*P* < 0.05 for all analyses).

**Table 1:** Patients' characteristics on admission and their relationship with red cell distribution width (RDW) stratified at median*†

	Overall (N = 3941)	RDW≤14% (N = 1943)	RDW>14% (N = 1998)	P
**Age** (years)	74 (64-82)	71 (60-80)	77 (67-84)	<0.001
**Male sex**	2215 (56.2)	1211 (62.3)	1004 (50.3)	<0.001
**Reason for admission**				Overall <0.001
Asymptomatic	92 (2.4)	42 (2.2)	52 (2.6)	0.364
Pneumonia	2665 (67.6)	1416 (72.9)	1249 (62.5)	<0.001
Temperature without pneum.	124 (3.1)	59 (3)	65 (3.3)	0.697
Acute medical condition	503 (12.8)	175 (9)	328 (16.4)	<0.001
Acute neurological condition	176 (4.5)	88 (4.5)	88 (4.4)	0.849
Acute surgical condition	379 (9.6)	163 (8.4)	216 (10.8)	0.001
**Day of disease on admission**	5 (1-9)	6 (2-10)	3 (1-8)	<0.001
**ECOG status on admission**	3 (1-4)	2 (1-3)	3 (2-4)	<0.001
**Pneumonia**	3485 (88.4)	3485 (88.4)	1756 (87.9)	0.281
**Oxygen therapy**	3226 (81.9)	1577 (81.2)	1649 (82.5)	0.264
**MEWS score**	2 (1-4)	2 (1-4)	2 (1-4)	0.357
**COVID-19 symptom severity**				0.006
Mild	423 (10.7)	198 (10.2)	225 (11.3)	0.278
Moderate	202 (5.1)	116 (6)	86 (4.3)	0.018 *
Severe	2730 (69.3)	1368 (70.4)	1362 (68.2)	0.128
Critical	586 (14.9)	261 (13.4)	325 (16.3)	0.012
**Other infection on admission**	576 (14.6)	199 (10.2)	377 (18.9)	<0.001
**Charlson comorbidity index**	4 (3-6)	4 (2-5)	5 (4-7)	<0.001
**Nm. of drugs in chr. therapy**	5 (3-8)	4 (2-7)	6 (4-9)	<0.001
**Arterial hypertension**	2741 (69.6)	1283 (66)	1458 (73)	<0.001
**Diabetes mellitus**	1185 (30.1)	547 (28.2)	638 (31.9)	<0.001
**Hyperlipoproteinemia**	944 (24.0)	433 (22.3)	511 (25.6)	<0.001
**Obesity**	1056 (26.8)	535 (27.5)	521 (26.1)	0.301
**Chr. heart failure**	638 (16.2)	190 (9.8)	448 (22.4)	<0.001
**Atrial fibrillation**	711 (18.0)	213 (11)	498 (24.9)	<0.001
**Coronary artery disease**	606 (15.4)	244 (12.6)	362 (18.1)	<0.001
**Previous CVI**	460 (11.7)	168 (8.6)	292 (14.6)	<0.001
**Previous myocardial inf.**	363 (9.2)	146 (7.5)	217 (10.9)	<0.001
**Chr. kidney disease**	486 (12.3)	121 (6.2)	365 (18.3)	<0.001
**COPD**	281 (7.1)	97 (5)	184 (9.2)	<0.001
**Chronic liver disease**	110 (2.8)	34 (1.7)	76 (3.8)	<0.001
**Liver cirrhosis**	49 (1.2)	4 (0.2)	45 (2.3)	<0.001
**Active malignancy**	419 (10.6)	90 (4.6)	329 (16.5)	<0.001
**Metastatic malignancy**	273 (6.9)	49 (2.5)	224 (11.2)	<0.001
**History of malignancy**	704 (17.9)	221 (11.4)	483 (24.2)	<0.001
**Dementia**	814 (20.7)	298 (15.3)	516 (25.8)	<0.001
**Alcohol use**	215 (5.5)	100 (5.1)	115 (5.8)	0.400
**Smoking**	230 (5.8)	110 (5.7)	120 (6)	0.645
**IL-6** (pg/ml)	53.4 (20.9-121.8)	47.1 (17.5-106)	64.3 (24.9-149.4)	0.003
**Procalcitonin** (ng/mL)	0.22 (0.09-0.76)	0.17 (0.08-0.48)	0.3 (0.12-1.11)	<0.001
**WBC** (x10^9^/L)	8 (5.7-11.2)	7.8 (5.7-10.8)	8 (5.7-11.6)	0.039
**Hemoglobin** (g/L)	128 (113-141)	135 (124-145)	119 (103-134)	<0.001
**MCV** (fL)	88.9 (85.6-92.2)	89.2 (86.6-92.2)	88.4 (84.5-92.3)	<0.001
**MCHC** (g/L)	333 (324-340)	336 (329-343)	329 (320-336)	<0.001
**Reticulocyte count** (x10^9^/L)	53 (36-72.5)	38.5 (33-55)	55 (41-82.5)	0.022
**Platelets** (x10^9^/L)	220 (163-296)	223 (170-294)	217 (155-298)	0.009
**CRP** (mg/L)	88.3 (39.4-150.8)	88.2 (37.3-148.4)	88.5 (40.8-152.2)	0.312
**Ferritin** (μg/L)	711 (386-1290)	808 (450-1384)	611 (310.5-1166.5)	<0.001
**D-dimers** (mg/L FEU)	1.42 (0.73-3.58)	1.15 (0.63-2.64)	1.75 (0.87-4.24)	<0.001
**eGFR** (ml/min/1.73 m^2^)	71.5 (45.8-90.3)	79.7 (58.6-93.3)	60.6 (34.6-85.6)	<0.001
**LDH** (U/L)	335 (248-454)	345.5 (254-463)	327 (242-443)	0.008
**AST** (U/L)	41 (18-64)	43 (30.8-66)	39 (26-61)	<0.001
**ALT** (U/L)	31 (19-52)	35 (23-59)	26 (16-44)	<0.001
**GGT** (U/L)	42 (24-82)	44 (26-79)	40 (22-84)	0.017
**ALP** (U/L)	72 (56-97)	66 (53-86)	79 (59-110)	<0.001
**Total bilirubin** (μmol/L)	11.4 (8.6-15.9)	11.3 (8.8-14.9)	11.6 (8.5-17.3)	0.044
**Albumin** (g/L)	32 (28-35)	33 (30-35)	30 (27-34)	<0.001
**PT** (%)	100 (89-109)	102 (93-110)	97 (85-107)	<0.001

*Abbreviations: RDW – red cell distribution width; IQR – interquartile range; ECOG – Eastern Cooperative Oncology Group; MEWS – Modified Early Warning Score; nm. – number; chr. – chronic; CVI – cerebrovascular insult; inf. – infarction; COPD – chronic obstructive pulmonary disease; WBC – white blood cells; MCV – mean corpuscular volume; MCHC – mean corpuscular hemoglobin concentration; CRP – C-reactive protein; eGFR – estimated glomerular filtration rate; LDH – lactate dehydrogenase; AST – aspartate aminotransferase; ALT – alanine aminotransferase; GGT – gamma-glutamyl transferase; ALP – alkaline phosphatase; PT – prothrombin time.

†Data are n (%) or median (interquartile range). For comparison of numerical and categorical variables between lower and higher RDW groups the Mann-Whitney U and the Χ^2^ or the Fisher test were used, respectively.

Charlson comorbidity index was significantly higher in patients with higher RDW, with higher RDW being associated with arterial hypertension, diabetes mellitus, hyperlipoproteinemia, chronic heart failure, atrial fibrillation, coronary artery disease, history of cerebrovascular insult, history of myocardial infarction, chronic kidney disease, chronic obstructive pulmonary disease, chronic liver disease, liver cirrhosis, active malignancy, metastatic malignancy and dementia (*P* < 0.05 for all analyses).

**Figure 1:**
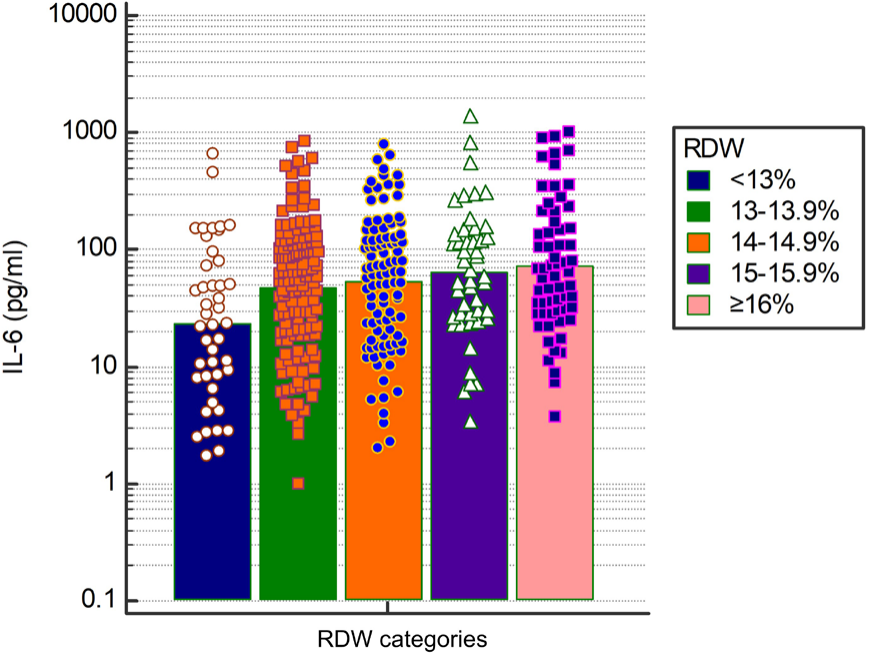
Median interleukin (IL)-6 values across red cell distribution width (RDW) percent point categories.

RDW was not significantly associated with C-reactive protein but was significantly associated with higher interleukin-6, which gradually increased with rising RDW percent points (*P* < 0.001 for trend of increase and *P* = 0.003 for overall difference in interleukin-6 between the categories) (Figure 1). Higher RDW was significantly associated with higher white blood cells, lower hemoglobin, lower MCV, lower mean corpuscular hemoglobin concentration, higher reticulocyte count, lower platelets, higher procalcitonin, lower ferritin, higher D-dimer, lower eGFR, lower lactate dehydrogenase, lower aspartate aminotransferase, lower alanine aminotransferase, lower gamma-glutamyl aminotransferase, higher alkaline phosphatase, higher total bilirubin, lower albumin, and lower prothrombin time (*P* < 0.05 for all analyses).

The associations of RDW with clinical outcomes during and after hospital stay

Associations of RDW with clinical outcomes are presented in Table 2. The median length of hospital stay was 10 days and did not significantly differ between patients with lower and higher RDW. Although patients with RDW>14% and ≤14% (cut-off optimized for survival discrimination) had similar frequencies of intensive care unit use, high flow oxygen therapy and mechanical ventilation use, arterial and venous thrombotic events, and bacteriemia, we established optimized cut-off points for specific outcomes with ROC curve analysis. Patients with RDW at the cut-off level of >13.1 were significantly more likely to need high-flow oxygen therapy (20.2% vs 16.4%; *P* = 0.021), mechanical ventilation (18.1% vs 11.7%; *P* < 0.001), and intensive care unit (23.9% vs 17.9%; *P* < 0.001). Patients with RDW>13.8% were significantly more likely to experience prolonged immobilization for >7 days without bathroom privileges (49.1% vs 38.3%; *P* < 0.001). Regarding venous thromboembolism, higher RDW was significantly associated with venous thrombotic events (6.7% vs 4.8% for RDW>14.9% vs ≤14.9%; *P* = 0.015) driven by a difference in the frequency of deep venous thromboses (3.3% vs 1.6% for RDW>14.8% vs ≤14.8%; *P* < 0.001). However, no relevant cut-off was established for pulmonary embolism or arterial thrombotic events. Regarding bleeding complications, higher RDW was significantly associated with documented bleeding (10.6% vs 6.8% for RDW>14.7% vs ≤14.7%; *P* < 0.001), namely bleeding from the gastrointestinal tract (6.7% vs 2.5% for RDW>15.5% vs ≤15.5%; *P* < 0.001) and major bleeding events (4.2% vs 2% for RDW>13.9% vs ≤13.9%; *P* < 0.001). Patients with RDW>12.9% compared with those with RDW≤12.9% were significantly more likely to experience bacterial sepsis (10.3% vs 6.7%; *P* = 0.017).

**Table 2:** Clinical outcomes of index coronavirus disease-2012 hospital stay and post-hospital discharge in relationship to red cell distribution width (RDW)^‡^

	Overall (N = 3941)	RDW≤14% (N = 1943)	RDW>14% (N = 1998)	P
**Length of hospitalization (days)**	10 (6-16)	10 (6-16)	10 (6-16)	0.329
**Intensive care unit**	901 (22.9)	448 (23.1)	453 (22.7)	0.774^†^
**High-flow oxygen therapy**	768 (19.5)	401 (20.6)	367 (18.4)	0.072^†^
**Mechanical ventilation**	669 (17)	314 (16.2)	335 (17.8)	0.179^†^
**Immobilization ≥7 days**	1753 (44.5)	760 (39.1)	993 (49.7)	<0.001*
**Venous thromboembolism**	212 (5.4)	102 (5.2)	110 (5.5)	0.713^†^
**Pulmonary embolism**	143 (3.6)	77 (4)	66 (3.3)	0.268
**Deep venous thrombosis**	85 (2.2)	35 (1.8)	50 (2.5)	0.129^†^
**Arterial thrombosis**	228 (5.8)	109 (5.6)	119 (6)	0.648
**Acute myocardial infarction**	67 (1.7)	30 (1.5)	37 (1.9)	0.455
**Acute cerebrovascular insult**	108 (2.7)	59 (3)	49 (2.5)	0.262
**Bleeding**	318 (8.1)	126 (6.5)	192 (9.6)	<0.001*
**Major bleeding**	126 (3.2)	42 (2.2)	84 (4.2)	<0.001*
**Gastrointestinal bleeding**	132 (3.3)	41 (2.1)	91 (4.6)	<0.001*
**Bacterial sepsis**	390 (9.9)	186 (9.6)	204 (10.2)	0.503^†^
**30-day survival rate, %**	65.6	75.7	55.8	<0.001*
**Hospital readmission** ^§^	76 (3)	33 (2.3)	43 (3.9)	0.016*
**6-month post-discharge survival rate**^§^, %	91.6	95.5	86.1	<0.001*

*Statistically significant at level *P* < 0.05.

†significant difference at level *P* < 0.05 on optimized RDW cut-off level (intensive care unit RDW>13.1%, high flow oxygen therapy RDW>13.1%, mechanical ventilation RDW>13.1%, venous thromboembolism RDW>14.9%, deep venous thrombosis RDW>14.8%, bacterial sepsis RDW>12.9%).

‡Data are n (%) or median (interquartile range), unless otherwise specified. For comparison of numerical and categorical variables between lower and higher RDW groups the Mann-Whitney U and the Χ^2^ or the Fisher test were used, respectively. Survival was compared by using the log-rank test.

§evaluated only in index hospitalization survivors (N = 2545).

**Figure 2:**
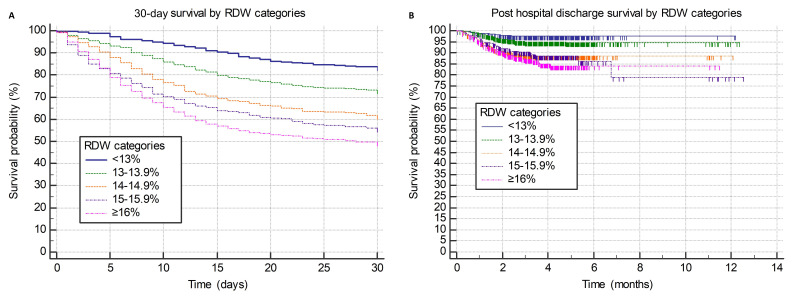
(**A**) Survival at 30-days from hospital admission and (**B**) post-hospital-discharge survival stratified according to the red cell distribution width (RDW) percent point categories.

Considering survival outcomes, patients with RDW>14% experienced a significantly lower 30-day survival rate (55.8% vs 75.7%; hazard ratio [HR] 2.14; *P* < 0.001) and a lower 6-month post-hospital discharge survival rate (86.1% vs 95.5%; HR 3.11; *P* < 0.001). With each rising percent point of RDW on admission, we observed gradually worsening 30-day survival and post-hospital discharge survival (Figures 2A and 2B). Patients having RDW 13%-13.9%, 14%-14.9%, 15%-15.9%, and ≥16% in comparison with those having RDW<13% had HR 1.79 (*P* < 0.001), HR 2.76 (*P* < 0.001), HR 3.42 (*P* < 0.001), and HR 4.12 (*P* < 0.001) for 30-day survival, respectively. They also had HR 2.03 (*P* = 0.056), HR 4.72 (*P* < 0.001), HR 4.78 (*P* < 0.001), and HR 6.06 (*P* < 0.001) for post-discharge survival, respectively.

In a series of multivariate Cox regression models, we demonstrated robust independent prognostic properties of RDW for survival. RDW remained significantly associated with 30-day survival in the Cox regression model adjusted for age, sex, COVID-19 symptom severity, and Charlson comorbidity index (RDW>14% HR = 1.62, *P* < 0.001; age HR = 1.04, *P* < 0.001; male sex HR = 1.2; *P* < 0.001; COVID-19 severity moderate vs mild *P* = 0.669; COVID-19 severity severe vs mild HR = 9, *P* > 0.001; COVID-19 severity critical vs mild HR = 22.63, *P* < 0.001; Charlson comorbidity index HR = 1.11; *P* < 0.001). Similarly, RDW remained significantly associated with post-hospital discharge mortality in the Cox regression model adjusted for age, sex, COVID-19 severity, and Charlson comorbidity index (RDW>14% HR = 2.01, *P* < 0.001; age HR = 1.05, *P* < 0.001; male sex *P* = 0.708; COVID-19 severity moderate vs mild *P* = 0.987; COVID-19 severity severe vs mild *P* = 0.523; COVID-19 severity critical vs mild HR = 2.5, *P* = 0.001; Charlson comorbidity index HR = 1.22; *P* < 0.001). RDW remained significantly associated with 30-day survival after adjusting for COVID-19 severity and COVID-19 prognostic scores CURB-65, 4C mortality, COVID gram, and VACO index (RDW>14% HR = 1.45; *P* < 0.001; COVID-19 severity categories, HR = 1.77, *P* < 0.001; CURB-65 categories HR = 1.39, *P* < 0.001; 4C mortality categories HR = 1.7, *P* < 0.001; COVID gram categories HR = 2.67, *P* < 0.001; VACO index categories HR = 3.51, *P* = 0.002).

## Discussion

Our study, the largest single-institution cohort of hospitalized COVID-19 patients investigated so far, showed a gradual worsening of 30-day and post-hospital discharge survival with each rising percent point of RDW on admission. In addition, our study was first to investigate the associations of RDW with various clinical outcomes and to assess the prognostic role of RDW in the context of established COVID-19 prognostic scores. We demonstrated that RDW possessed independent prognostic properties and had a good potential for improvement of prognostication of hospitalized COVID-19 patients.

The main cause of RDW elevation varies from patient to patient with the same disease. This cause is hard to determine since it can be an impaired function of a number of organ systems (5,19). Nevertheless, RDW seems to be a universal marker of biological fitness as it reflects unwanted clinical outcomes in all diseases where it was investigated. Our data show that RDW is profoundly affected by the presence of prior comorbidities and age, with patients with higher RDW being more likely to have chronic metabolic comorbidities, more unfavorable cardiovascular risk profile, chronic renal disease, chronic obstructive pulmonary disease, liver cirrhosis, active and metastatic malignancy, and dementia. Interestingly, patients with higher RDW significantly grouped only among patients with critical COVID-19 on admission, and no similar association of RDW was observed with either radiological presence of pneumonia or the need for oxygen supplementation. Thus, the mentioned chronic comorbidities might predispose to higher functional impairment and critical clinical presentation observed in our study. Despite similar frequency of pneumonia and respiratory insufficiency at baseline, patients with higher RDW were significantly more likely to develop respiratory deterioration during hospital stay and to need intensive care and more advanced respiratory support.

RDW has a complex relationship with COVID-19-associated inflammatory state. Despite a significant association with higher interleukin-6 concentrations, which increased with an increase in RDW percent points, RDW was not significantly associated with CRP concentration on admission. In addition, RDW was significantly associated with higher WBC, higher procalcitonin, higher D-dimer, lower albumin, lower prothrombin time, and higher bilirubin on admission, but also with lower liver transaminases, lower ferritin, and lower lactate dehydrogenase. This ambiguous inflammatory profile associated with higher RDW further implies that RDW elevation is affected by mechanisms other than acute inflammatory state associated with COVID-19. The magnitude of RDW elevation occurring in acute COVID-19 infection is unknown, and high RDW, and consequently higher hazard of death, might represent a higher degree of chronic inflammation, a higher comorbidity burden, and a predisposition to worse clinical outcomes predating acute COVID-19. It was speculated whether patients with higher RDW might present later during disease course and thus represent more advanced inflammation (20). Our data suggest the opposite – patients with higher RDW were more likely to present earlier during disease course, and RDW might be elevated because of acutization of chronic medical conditions other than pneumonia due to active COVID-19 infection.

A number of studies and several large meta-analyses have confirmed potent prognostic properties of RDW for the survival of COVID-19 patients (13-16). Our study provides important novelty as it comprehensively assesses several important clinical outcomes and investigates how different levels of RDW increase reflect on survival, both in univariate and multivariate context controlling for clinically relevant parameters. RDW on admission not only predicts short- and long-term survival but also predicts a wide range of unwanted clinical outcomes, such as the need for more intensive respiratory support, prolonged immobilization, deep venous thrombosis, bacterial sepsis, and bleeding. The ability of RDW to discriminate between different outcomes changes when using different cut-off levels. Lower cut-off values optimally predicted complications of infectious diseases (bacterial sepsis and tendency for COVID-19 deterioration), whereas higher values optimally predicted venous thrombotic events. Although pronounced anisocytosis reflects a worse prognosis for thrombotic and septic outcomes in COVID-19 patients, the precise mechanism underlying these processes is not completely clear (compared with the intuitive role of some other blood components such as platelets in the same events [21]). Considering survival as the most important outcome, long-term and short-term prognostic properties of RDW are independent from COVID-19 severity on admission, age, and comorbidities. Additionally, RDW covers different parts of prognostic spectrum than WHO-defined COVID-19 severity categories: CURB-65, 4C mortality, COVID gram, and VACO index. Therefore, RDW might be very useful in assessing the prognosis of hospitalized COVID-19 patients. Our results need to be validated by future studies in independent samples and by using RDW to further extend prognostic scores.

Limitations of our study are single-center experience, retrospective study design, and the lack of longitudinal assessment of RDW values. Our results are representative of tertiary level institution, with a majority of patients being elderly, having severe or critical COVID-19, and having high comorbidity burden. The main strength of our study is a large sample of patients who were uniformly exposed to standardized diagnostic and therapeutic procedures. This gives the study high statistical power to assess clinical and prognostic associations of RDW in the context of hospitalized COVID-19 patients.

In conclusion, RDW has a complex relationship with COVID-19-associated inflammatory state and is profoundly affected by prior comorbidities. It has a good potential for improvement of prognostication of hospitalized COVID-19 patients.
